# The Bathtub Illusion

**DOI:** 10.1177/2041669519853594

**Published:** 2019-07-04

**Authors:** Marco Bertamini

**Affiliations:** Department of Psychological Sciences, University of Liverpool, UK

**Keywords:** visual illusion, size-distance, phenomenological method, perception of size

## Abstract

When a person looks at the fingers of their own hand as they line up in depth, the impression may emerge that the little fingers, which are farther away, are located too far and if so they are not part of the same hand. I describe the conditions and suggest this is due to the size difference between fingers (size-distance scaling). A role of size on perceived distance here is more powerful than knowledge about our own body.

As we concentrate, we often touch the tip of the fingers of the left and right hands, matching the corresponding fingers so that the index touches the index and so on. Figure 1(a) illustrates this type of situation. As the little fingers are shorter than the others, they end up slightly separated. When looking directly at the little fingers, I have the impression that they are too far and therefore are not part of the same hand. The effect is present in binocular or monocular vision. Binocularly one can fixate the little fingers (so that the other fingers have crossed disparity) or fixate the other fingers (so that the little fingers have uncrossed disparity). It also works in an image as in Figure 1(a). You can try all these conditions with your hands right now anywhere (except if you are driving a car, but then you should not be reading either).

**Figure 1.**
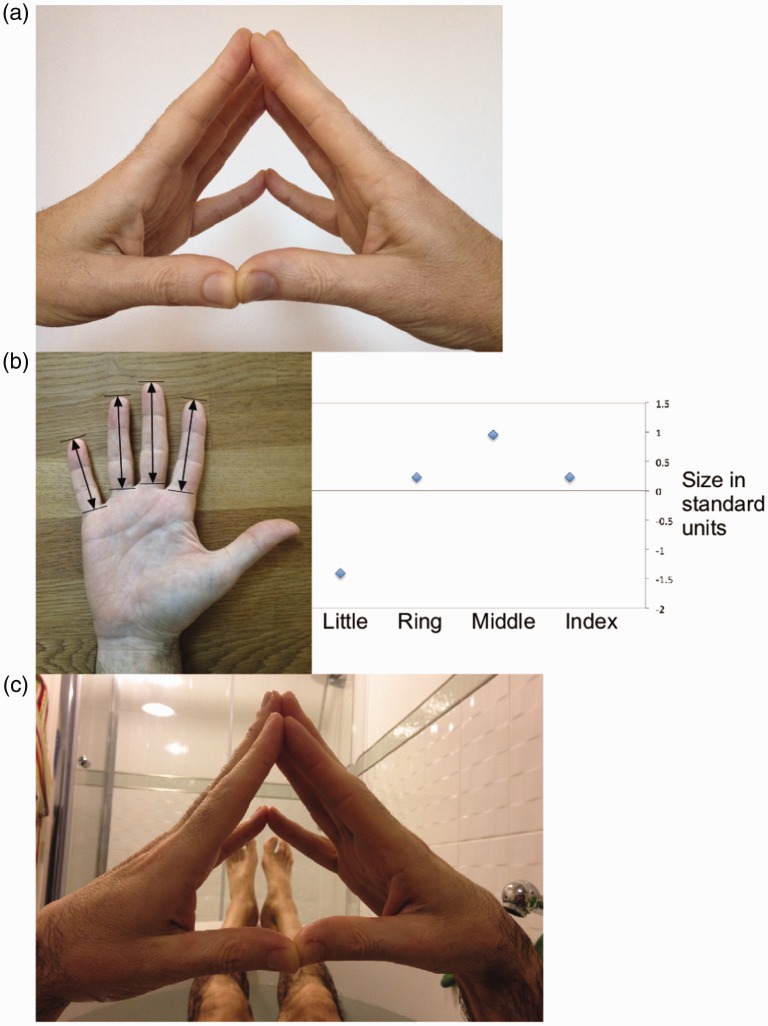
(a) A close-up photo of the hands. The little fingers do not convincingly belong to the hand and may appear to be detached. (b) For most hands, the little finger is shorter than index, middle, and ring fingers, thus it is known as the *little* finger. (c) The image of the hands at the time of the Eureka experience.

To establish whether other people shared my experience, I printed the photograph in Figure 1(a) and asked 10 naive observers (five females and five males) to position their own fingers as in the image. The approach was phenomenological (Kubovy, 1999; Vicario, 1993). One person said that there was no interesting effect at all. Of the other nine observers, two were not entirely sure, and an effect of the fingers perceived as too far was clear to 7 out of 10 people. Excluding only the person who did not see any illusion at all, for five observers, the effect was clearer in monocular vision; for other three, monocular or binocular vision made no difference, and for one, it was stronger in binocular vision. When asked to compare the view of their own hands with the photograph, six observers reported that they could see a stronger effect in the photograph, two with the hands, and one said that it was the same.

It is likely that this illusion is a consequence of the fact that size is associated with perceived distance. In the case of my right hand, I took the lengths from the tip to the Metacarpo-phalangeal crease, as shown in Figure 1(b). The approximate values were index: 7.7 cm, middle: 8.4 cm, ring finger: 7.7 cm, and little finger: 6.1 cm. Therefore, the little finger is an outlier as it is 1.4 standard deviations away from the mean (of this small sample). In a large sample of men (*N* = 250), average length for index and ring fingers were 7.8 cm and 8.1 cm, respectively (Aboul-Hagag, Mohamed, Hilal, & Mohamed, 2011). I could not find a value for the little finger, but from these values, it seems that my hand is close to an average male hand.

This illusion is a bit like the Ames window illusion: the shorter side of the trapezoid is perceived as farther away because of the link size/distance. Unlike the Ames window, however, this effect illustrates how this factor overcomes expectations and knowledge about our own body. Other illusions have demonstrated the power of depth factors to win over expectations, for example, this is the case for the effect of visual completion demonstrated by Ekroll, Sayim, Van der Hallen, and Wagemans (2016). In their illusion, the index finger is perceived as shorter than the other fingers. Even in the case of the Ames window, holding the rigid model of the window in your arms will make one arm feel longer than the other (Bertamini & Bruno, 2008). A secondary factor may be the t-junction forming between the hand and the little fingers, at least from some angles.

The main interest in this illusion comes from its simplicity and the fact that it can be demonstrated without additional materials in a classroom. Based on a small sample of 10 people, it appears that a majority can experience the effect. A phenomenological approach is necessary, and it is important to go beyond one’s expectations (we know the fingers are attached to the hand). It is also important to allow time and not consider only the first impression. These are aspects of the phenomenological approach (Bianchi & Davies, 2019), and the illusion may have a didactic role in relation to phenomenology.

Because other illusions exist that affect fingers (as in Ekroll et al., 2016) and because of the old and well-known tricks of the detached finger, I am calling this the bathtub illusion from the location of its discovery. The term *stretching out in the tub* was used in 2010 for an entry to the illusion of the year contest by Lydia Maniatis (http://illusionoftheyear.com/cat/top-10-finalists/2010/), but to the best of my knowledge, that effect is not known as the bathtub illusion. In Figure 1(c), we can see the illusion in the original context, which I include for full disclosure (only in the sense of historical context, you will be glad to see).
